# Application of activated barrier hopping theory to viscoplastic modeling of glassy polymers

**DOI:** 10.1007/s11043-017-9369-5

**Published:** 2017-10-30

**Authors:** J. Sweeney, P. E. Spencer, D. Vgenopoulos, M. Babenko, F. Boutenel, P. Caton-Rose, P. D. Coates

**Affiliations:** 1 School of Engineering, Faculty of Engineering and Informatics, University of Bradford, Bradford, BD7 1DP UK; 2 Institut Clement Ader (ICA); Universite de Toulouse; CNRS, IMT Mines Albi, INSA, ISAE-SUPAERO, UPS; Campus Jarlard, F-81013 Albi, France

**Keywords:** Polymer, Viscoplastic, Constitutive model, Finite element analysis

## Abstract

An established statistical mechanical theory of amorphous polymer deformation has been incorporated as a plastic mechanism into a constitutive model and applied to a range of polymer mechanical deformations. The temperature and rate dependence of the tensile yield of PVC, as reported in early studies, has been modeled to high levels of accuracy. Tensile experiments on PET reported here are analyzed similarly and good accuracy is also achieved. The frequently observed increase in the gradient of the plot of yield stress against logarithm of strain rate is an inherent feature of the constitutive model. The form of temperature dependence of the yield that is predicted by the model is found to give an accurate representation. The constitutive model is developed in two-dimensional form and implemented as a user-defined subroutine in the finite element package ABAQUS. This analysis is applied to the tensile experiments on PET, in some of which strain is localized in the form of shear bands and necks. These deformations are modeled with partial success, though adiabatic heating of the instability causes inaccuracies for this isothermal implementation of the model. The plastic mechanism has advantages over the Eyring process, is equally tractable, and presents no particular difficulties in implementation with finite elements.

## Introduction

Solid polymers are mechanically nonlinear, time-dependent and capable of attaining large deformations, especially in processing regimes. Progress has been made in recent years in understanding their stress-strain behavior, and in implementing complex constitutive equations that reflect experimental observations. A viscoplastic approach is customarily adopted. At the heart of such a model there is a plastic mechanism, in which the stress must be nonlinearly dependent on strain rate to give a realistic representation for polymers.

The plastic mechanism most frequently used in polymer modeling is the Eyring process (Halsey et al. 1945). It has been shown to provide useful models of creep (Mindel and Brown 1973; Wilding and Ward 1978, 1981), strain-rate-dependent yield (Duckett et al. 1978; Liu and Truss 1994; Buckley and Jones 1995), and stress relaxation (Sweeney and Ward 1990; Sweeney et al. 2012, 2014). The modeling of stress-strain behavior, stress relaxation and yield is often accomplished by combining the Eyring process with one or more elastic elements, as pioneered by Haward and Thackray (1968).

The Eyring process is used because it has a number of attractive features. For strain-rate-dependent yielding, the Eyring process predicts an Arrhenius-type relation, in which yield stress varies linearly with the logarithm of rate. This is in many instances a satisfactory approximation. For stress relaxation, it provides a simple expression for the time dependence of stress via the analysis of Guiu and Pratt (1964), which has been shown to fit well to polymer behavior (Sweeney and Ward 1990) but not in all circumstances (Sweeney et al. 2012, 2014). There are also some drawbacks. A significant one is that the Arrhenius relation for yield stress does not necessarily apply over wide ranges of strain rate. This has motivated Eyring-based models of greater complexity, which include a minimum of two processes acting in parallel (Ree and Eyring 1955; Roetling 1965; Wilding and Ward 1978, 1981; Truss et al. 1981; Foot et al. 1987; Sweeney et al. 2012).

These concerns have been discussed by Chen and Schweizer who have proposed an alternative mechanism for glassy polymers in a series of publications (Chen and Schweizer 2007a, 2007b, 2008, 2011; Riggleman et al. 2008). The aim of this paper is to develop constitutive models based around this new mechanism that can be implemented numerically, with a view to their future inclusion in finite element codes. The approach adopted is to combine the plastic mechanism with elastic elements, leading to essentially a viscoplastic approach, rather than the viscoelastic framework adopted by Chen and Schweizer (2008). This approach has been shown to be applicable to yielding of PVC for a very wide range of strain rates up to impact speeds, and to stress relaxation of polycarbonate (Sweeney and Spencer 2015). Here we extend the model application to temperature-dependent yield and demonstrate its implementation in finite element modeling.

## Modeling

Plastic deformation is assumed to be associated with shear stress and shear strain only, so that there is no volume change and the hydrostatic component of stress has no influence. Central to this model is a plastic mechanism, in which shear stress $\tau$ and plastic shear strain rate $\dot{\gamma}$ are related by Chen and Schweizer (2007a):

1 $$ \tau ( T ) = \tau_{\mathrm{abs}} ( T ) \biggl[ 1 - \biggl( \frac{ - kT\ln ( \dot{\gamma} \tau_{0} ) - \varepsilon}{a_{c}F_{B}(T)} \biggr)^{h} \biggr] , $$

where $\tau_{\mathrm{abs}}$ represents an absolute upper limit to the stress, $T$ is the absolute temperature, $k$ Boltzmann’s constant and $F_{B}$ is an energy barrier. $\tau_{0}$, $a_{c}$ and $\epsilon$ are amenable to direct physical interpretation, whereas $h$ is a fitting exponent arising from a power-law representation of the energy barrier as a function of stress. $h$ was given the value 0.4 by Chen and Schweizer (2007a), based on calculations for PMMA. For simplicity we rewrite Eq. (1):

2 $$ \tau = \tau_{\mathrm{abs}} \bigl[ 1 - \bigl( A\ln ( C\dot{\gamma} ) - D \bigr)^{h} \bigr] . $$

It is clear in both Eqs. (1) and (2) that there is no possibility for the shear rate $\dot{\gamma} $ to be zero. Correspondingly a zero shear stress $\tau$ is obtained for a nonzero shear rate. This is of no practical significance as long as this nonzero shear strain rate is small enough; it is analogous to the procedure adopted, when using an Eyring process, of approximating the hyperbolic sine function with an exponential (Ward and Sweeney 2013). Expressions for $A$, $C$ and $D$ can be deduced by comparing Eqs. (1) and (2). To apply this model in engineering problems we identify $\tau$ with the octahedral shear stress and $\dot{\gamma} $ with the scalar shear strain rate, defined by Ward and Sweeney (2013)

3 $$ \tau = \frac{1}{3} \bigl( (\sigma_{\mathrm{I}} - \sigma_{\mathrm{II}})^{2} + (\sigma_{\mathrm{II}} - \sigma_{\mathrm{III}})^{2} + ( \sigma_{\mathrm{III}} - \sigma_{\mathrm{I}})^{2} \bigr)^{1/2} $$

for principal stresses $\sigma_{\mathrm{I}}, \sigma_{\mathrm{II}}$ and $\sigma_{\mathrm{III}}$, and

4 $$ \dot{\gamma} = \bigl( \bigl(\dot{e}_{\mathrm{pI}}^{2} + \dot{e}_{\mathrm{pII}}^{2} + \dot{e}_{\mathrm{pIII}}^{2} \bigr)/3 \bigr)^{1/2} $$

for principal true plastic strain rates $\dot{e}_{\mathrm{pI}}, \dot{e}_{\mathrm{pII}}$ and $\dot{e}_{\mathrm{pIII}}$.

### Uniaxial deformations

In this paper some of the application of this model is to uniaxial deformations. Then shear stress and shear strain are simply related to tensile stress and strain, respectively. Conditions are defined as

5 $$ \sigma_{\mathrm{I}} = \sigma, \quad \quad \sigma_{\mathrm{II}} = \sigma_{\mathrm{III}} = 0 $$

and, assuming an incompressible plastic deformation,

6 $$ \dot{e}_{\mathrm{pII}} = \dot{e}_{\mathrm{pIII}} = - \frac{1}{2} \dot{e}_{\mathrm{pI}} = - \frac{1}{2}\dot{e}_{\mathrm{p}}. $$

Using Eqs. (3)–(6) in Eq. (2) gives the relation for uniaxial stretching along I:

7 $$ \sigma = \frac{3}{\sqrt{2}} \tau_{\mathrm{abs}} \bigl[ 1 - \bigl( A\ln (C \dot{e}_{p}/\sqrt{2} ) - D \bigr)^{h} \bigr]. $$

**Fig. 1:**
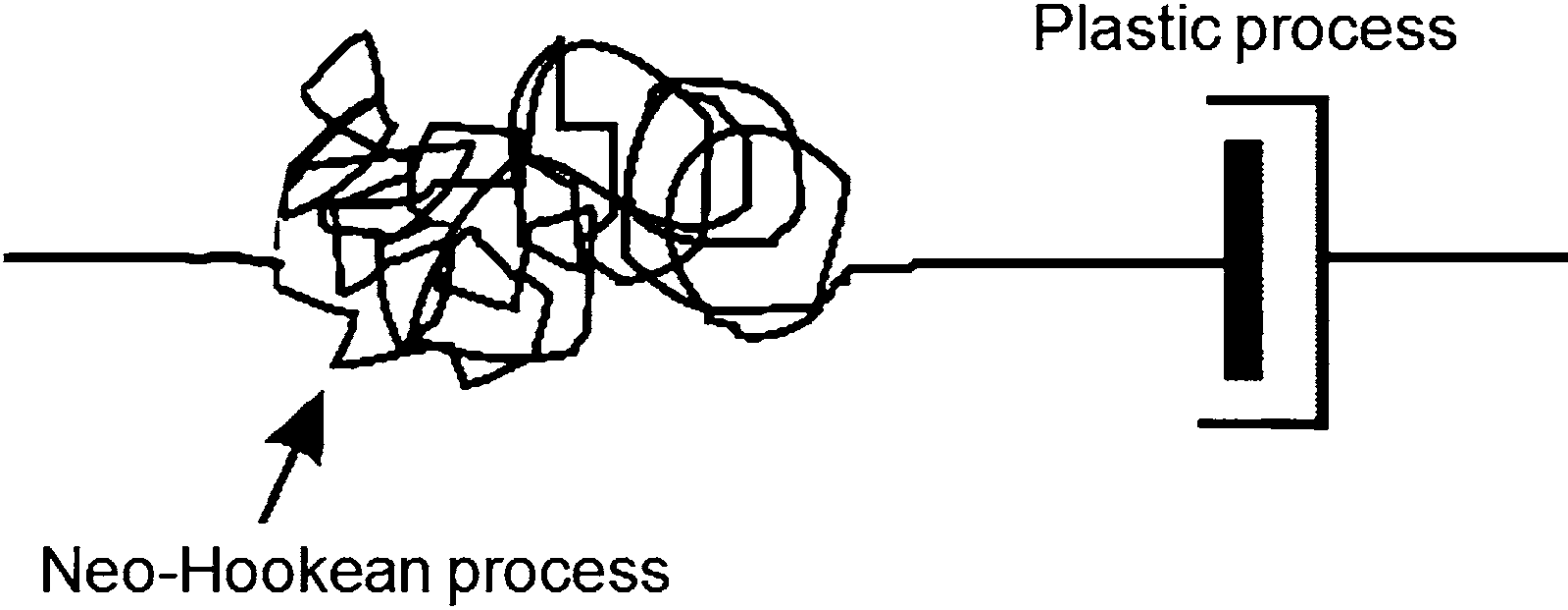
Nonlinear Maxwell-type model

Following the method of Sweeney and Spencer (2015), a constitutive model is created by adding an elastic element in series with this plastic mechanism, to give a Maxwell-like form of model illustrated in Fig. 1. Adopting a large deformation formulation, and using an incompressible Neo-Hookean form for the elastic element, then, for an elastic extension ratio $\lambda_{e}$, equilibrium requires that

8 $$ \frac{3}{\sqrt{2}} \tau_{\mathrm{abs}} \biggl[ 1 - \biggl( A\ln \biggl\{ \frac{C}{\sqrt{2}} \frac{\dot{\lambda}_{p}}{\lambda_{p}} \biggr\} - D \biggr)^{h} \biggr] - G \bigl( \lambda_{e}^{2} - 1/\lambda_{e} \bigr) = 0 , $$

where $G$ is the strength of the neo-Hookean mechanism. The plastic strain rate $\dot{e}_{p}$ has been expressed in terms of the plastic extension ratio $\lambda_{p}$ and its time derivative $\dot{\lambda}_{p}$. For a total extension ratio $\lambda$ on the model, $\lambda_{e} = \lambda/\lambda_{p}$ and Eq. (8) becomes

9 $$ \frac{3}{\sqrt{2}} \tau_{\mathrm{abs}} \biggl[ 1 - \biggl( A\ln \biggl\{ \frac{C}{\sqrt{2}} \frac{\dot{\lambda}_{p}}{\lambda_{p}} \biggr\} - D \biggr)^{h} \biggr] - G \bigl( \lambda^{2}/\lambda_{p}^{2} - \lambda_{p}/\lambda \bigr) = 0. $$

Equation (9) is solved numerically with time-marching $\lambda$ to give $\lambda_{p}$ and thus the stress $\sigma$, the latter via the neo-Hookean relation

10 $$ \sigma = G \bigl( \lambda^{2}/\lambda_{p}^{2} - \lambda_{p}/\lambda \bigr). $$

The model is capable of predicting stress-strain behavior, yield and stress relaxation by defining appropriate time histories for $\lambda$.

### Two-dimensional deformations

Here we outline a two-dimensional plane stress approach. This was implemented within finite element analyses using the package ABAQUS, with the material model incorporated via a ‘UMAT’ user-defined subroutine. The kinematics are similar to those used previously for high temperature stretching of polypropylene (Sweeney et al. 2009), polypropylene and polycarbonate (Sweeney et al. 2007) and fracture of polyethylene (Naz et al. 2010).

At each computed point the deformation is input to the subroutine in the form of the deformation gradient tensor $\mathbf{F}$ defined in global 1–2 axes. This strain corresponds to the total for the model of Fig. 1. There have been a number of approaches to the analysis of elastic-plastic behavior at large deformation, a useful summary of which has been made available by Figiel and Buckley (2009). Following the method that they classify as approach II, we split the deformation gradient $\mathbf{F}$ multiplicatively into elastic and plastic components, respectively, $\mathbf{F}^{e}$ and $\mathbf{F}^{p}$:

11 $$ \mathbf{F} = \mathbf{F}^{e}\mathbf{F}^{p}. $$

$\mathbf{F}^{p}$ is thus split into pure deformation $\mathbf{V}^{p}$ and rigid body rotation $\mathbf{R}$ (via the use of polar decomposition) to give

12 $$ \mathbf{F}^{p} = \mathbf{V}^{p}\mathbf{R} $$

while the elastic deformation gradient is symmetric, with $\mathbf{F}^{e} = \mathbf{V}^{e}$, so that Eq. (11) becomes

13 $$ \mathbf{F} = \mathbf{V}^{e}\mathbf{V}^{p}\mathbf{R} . $$

The principal values of $\mathbf{V}^{e}$ are the principal elastic extension ratios $\lambda_{\mathrm{I}}^{e}$ and $\lambda_{\mathrm{II}}^{e}$. Under incompressibility and plane stress conditions, the neo-Hookean relation gives principal stresses $\sigma_{i}$ of the stress tensor $\boldsymbol{\Sigma} $ as

14 $$ \sigma_{i} = G \bigl( \bigl( \lambda_{i}^{e} \bigr)^{2} - \bigl( \lambda_{\mathrm{I}}^{e} \lambda_{\mathrm{II}}^{e} \bigr)^{ - 2} \bigr)\quad (i = \mathrm{I}, \mathrm{II}) $$

and

15 $$ \sigma_{\mathrm{III}} = 0. $$

Equilibrium ensures that the stress tensors in both elastic and plastic elements of the model in Fig. 1 are equal. As in the uniaxial case above, the plastic strain rate is driven by the octahedral shear stress $\tau$ of Eq. (3) via Eq. (2), which can be rearranged as

16 $$ \dot{\gamma} = \frac{1}{C}\exp \bigl[ \bigl\{ ( 1 - \tau / \tau_{\mathrm{abs}} )^{1/h} + D \bigr\} /A \bigr]. $$

In two or three dimensions, the directional components of the strain rate need to be specified as proportions of the scalar rate $\dot{\gamma} $ via the use of a flow rule. Here we use the Levy–Mises flow rule (Ward and Sweeney 2013), for which the plastic strain rate components are proportional to the components of the deviatoric stress tensor $\boldsymbol{\tau}$ defined as

17 $$ \boldsymbol{\tau} = \boldsymbol{\Sigma} - \bar{\boldsymbol{\sigma}} \mathbf{I} , $$

where

18 $$ \bar{\boldsymbol{\sigma}} = \frac{1}{3}\operatorname{tr} ( \boldsymbol{\Sigma} ). $$

The plastic strain rate tensor $\mathbf{Q}$ is defined in terms of the plastic deformation $\mathbf{V}^{p}$ as

19 $$ \mathbf{Q} = \dot{\mathbf{V}}^{p}\mathbf{V}^{p - 1}. $$

Then the Levy–Mises flow rule may be expressed as

20 $$ \frac{\mathbf{Q}}{\dot{\gamma}} = \frac{\boldsymbol{\tau}}{\tau} . $$

An incremental approach is used, with strain rate assumed to be constant during each time increment. The current plastic stretch $V^{p}$ is related to the plastic strain $\mathbf{V}_{0}^{p}$ at the end of the previous time increment and the increment of plastic strain $\boldsymbol{\Delta}\mathbf{V}^{p}$ developed during the current increment by

21 $$ \mathbf{V}^{p} = \boldsymbol{\Delta} \mathbf{V}^{p}\mathbf{V}_{0}^{p} $$

which is related to Eq. (19) by

22 $$ \dot{\mathbf{V}}^{p} = \boldsymbol{\Delta} \mathbf{V}^{p}/\Delta t $$

for a time increment $\Delta t$. At the end of the time increment the deformation gradient is given by

23 $$ \mathbf{F} = \mathbf{V}^{e}\boldsymbol{\Delta} \mathbf{V}^{p}\mathbf{V}_{{0}}^{p} \mathbf{R}. $$

The values of $\mathbf{V}^{e}$ and $\boldsymbol{\Delta} \mathbf{V}^{p}$ are derived via an iterative process, to impose the condition that the stresses in the neo-Hookean element defined by Eq. (14) are equal to the stresses in the plastic mechanism governed by Eqs. (16)–(22), while the strains in the two elements are related to the total deformation gradient by Eq. (23). The iterative process begins with an input of $\mathbf{F}$ into the subroutine. An initial value of $\mathbf{V}^{e}$ is obtained on the basis that there is no increment in plastic strain, to give values of stress tensor $\boldsymbol{\Sigma} $ from Eq. (14). Plastic strain increments are obtained via Eqs. (16)–(22), to give an updated value of $\mathbf{V}^{e}$ from Eq. (23) to drive the next iteration. The process ends with the equilibrium of principal stresses between the neo-Hookean and plastic mechanisms.

### Temperature dependence of yield

Chen and Schweizer’s theory predicts a form for the temperature dependence of $A$. To find this form, observe that comparison of Eqs. (1) and (2) gives

24 $$ A = \frac{ - kT}{a_{c}F_{B} ( T )}. $$

$F_{B}$ is associated with the segmental hopping time $\tau_{\alpha} $ by the expression (Riggleman et al. 2008; Chen and Schweizer 2011)

25 $$ \tau_{\alpha} (T) = \tau_{0}\exp \biggl( \frac{\varepsilon_{A}}{kT} \biggr)\exp \biggl( \frac{a_{c}F_{B}(T)}{kT} \biggr). $$

It follows that

26 $$ \frac{a_{c}F_{B}(T)}{kT} = \ln \biggl( \frac{\tau_{\alpha} (T)}{\tau_{0}} \biggr) - \frac{\varepsilon}{kT}. $$

According to Chen and Schweizer (2007a), the quantity $\ln ( \frac{\tau_{\alpha} (T)}{\tau_{0}} )$ is a linear function of the temperature ratio $T_{g}/T$ with the slope only varying weakly with stress. Equation (26) is therefore rewritten as

27 $$ \frac{a_{c}F_{B}(T)}{kT} = \alpha + \beta \biggl( \frac{T_{g}}{T} \biggr) - \frac{\varepsilon}{kT} $$

for constants $\alpha$ and $\beta$. It follows from Eqs. (24) and (27) that

28 $$ A = - \biggl( \alpha + \beta \biggl( \frac{T_{g}}{T} \biggr) - \frac{\varepsilon}{kT} \biggr)^{ - 1} $$

or alternatively

29 $$ A = ( P + Q/T )^{ - 1} $$

for constants $P$ and $Q$.

## Historical data for temperature- and rate-dependent yield

The model of Fig. 1 for uniaxial conditions as defined in Eqs. (9) and (10) above is applied to the classic work on yield of polyvinylchloride by Bauwens-Crowet et al. (1969). The temperature range studied is 238–333 K, and the range of tensile strain rate covered is $2 \times 10 ^{-5}\mbox{--}2 \times 10 ^{-1}~\mbox{s}^{-1}$.

**Fig. 2:**
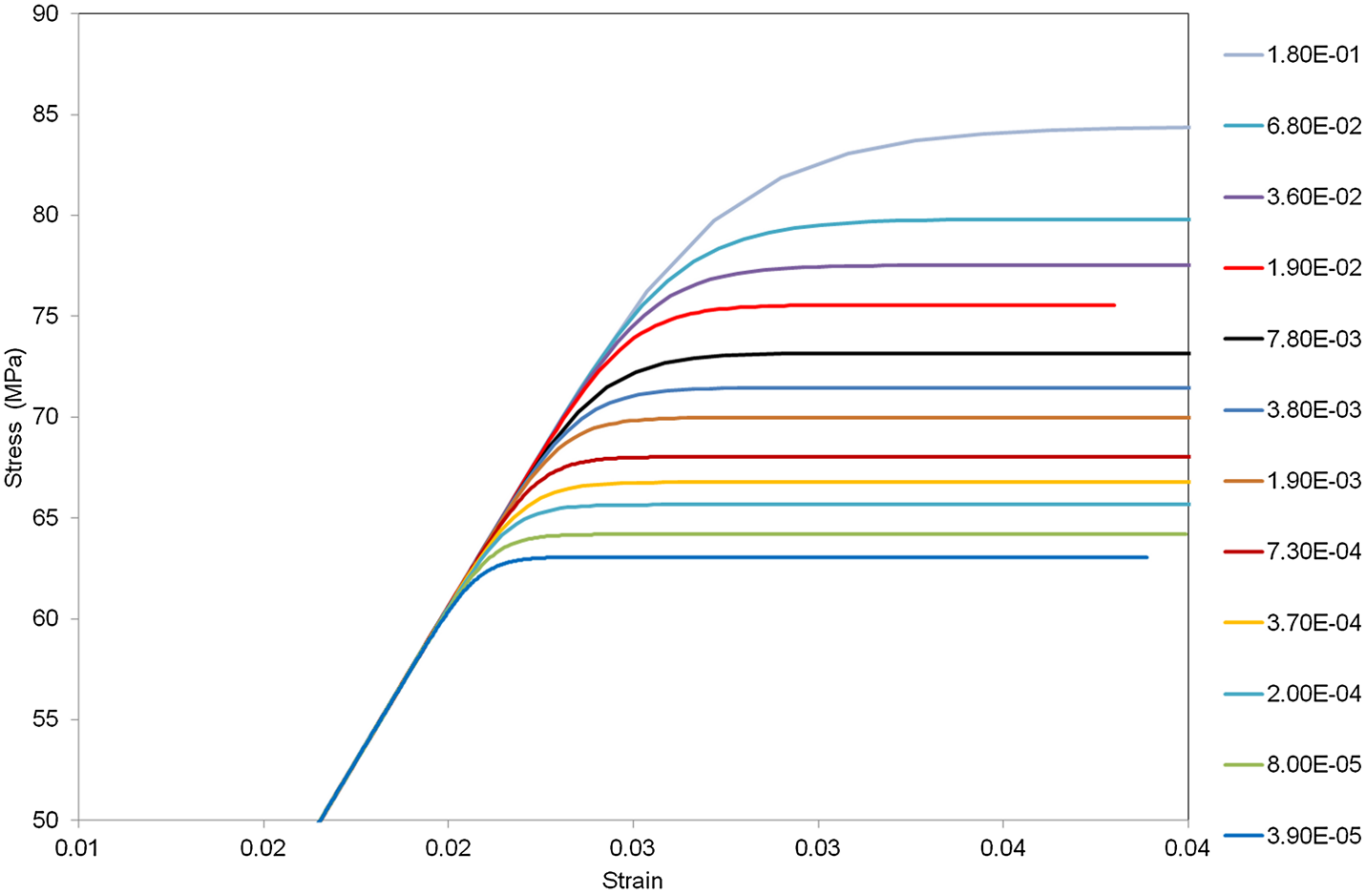
Stress-strain predictions for 273 K. Numbers in key refer to strain rates in $\mbox{s}^{-1}$

The model was subject to a constant total true strain rate $\dot{\lambda} /\lambda$ until a yield stress was attained. Model stress-strain curves are shown in Fig. 2 for the temperature 273 K. Yield stress is easily identified as the maximum.

The model parameters that are present in Eq. (9) are $\tau_{\mathrm{abs}}$, $A$, $C$, $D$, $h$ and $G$. The last, the hyperelastic constant $G$, has no effect on the yield stress and was arbitrarily assigned the value 1 GPa. Software was devised that calculated stress up to the yield point at each experimental strain rate, using Eqs. (9) and (10), for each temperature. For each temperature yield stresses were generated as a function of strain rate and the function

30 $$ E = \sum_{i = 1}^{N} \bigl( \sigma_{i}^{t}/\sigma_{i}^{e} - 1 \bigr)^{2} $$

was minimized, where $N$ is the number of data points and $\sigma_{i}^{t}$ and $\sigma_{i}^{e}$ are, respectively, the theoretical and experimental values of yield stress at the $i$th strain rate.

**Fig. 3:**
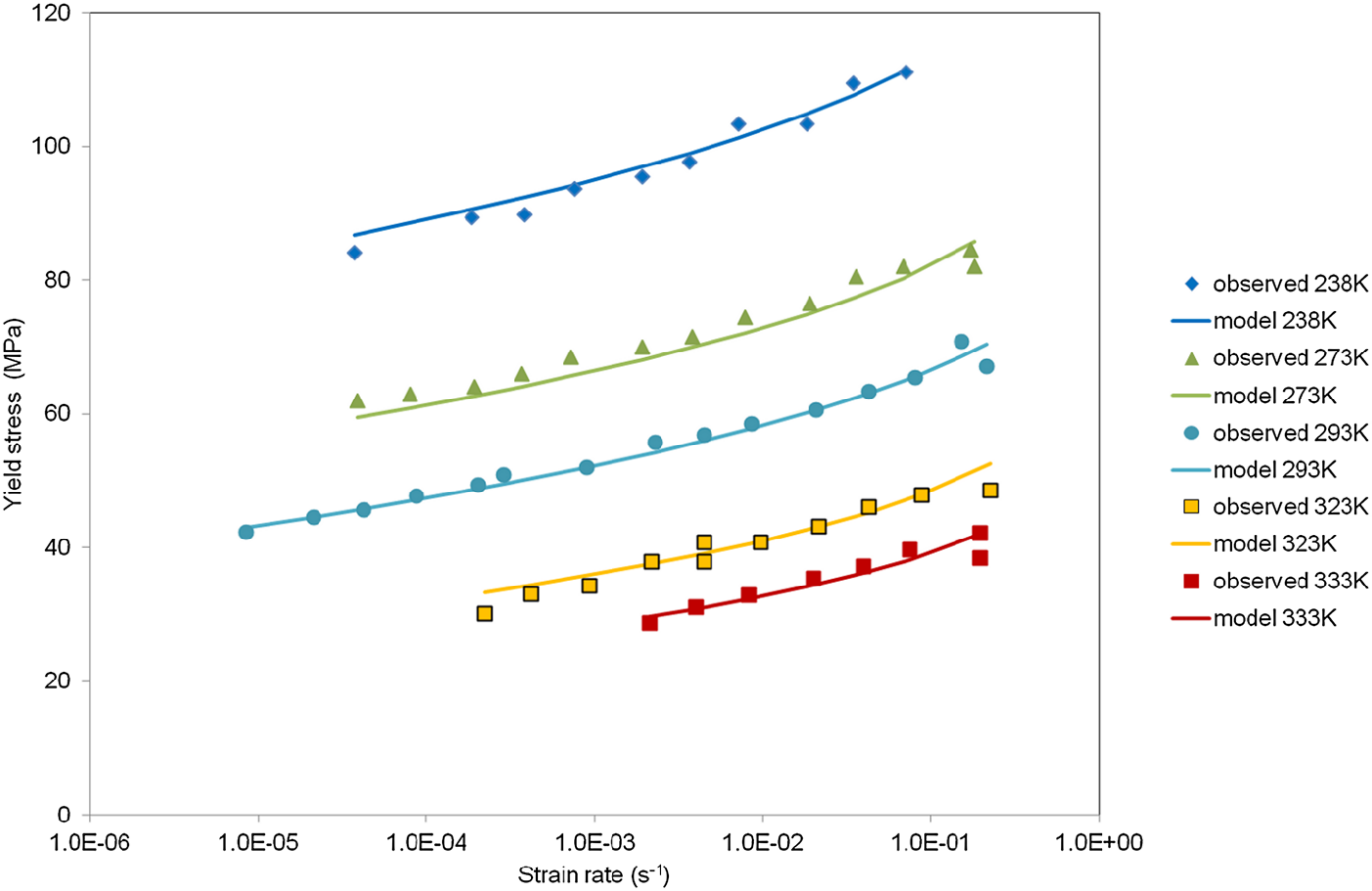
Observed results are those of Bauwens-Crowet et al. (1969). Model is that defined by Eqs. (9) and (10), with parameter values defined in Table 1 and Fig. 4

**Fig. 4:**
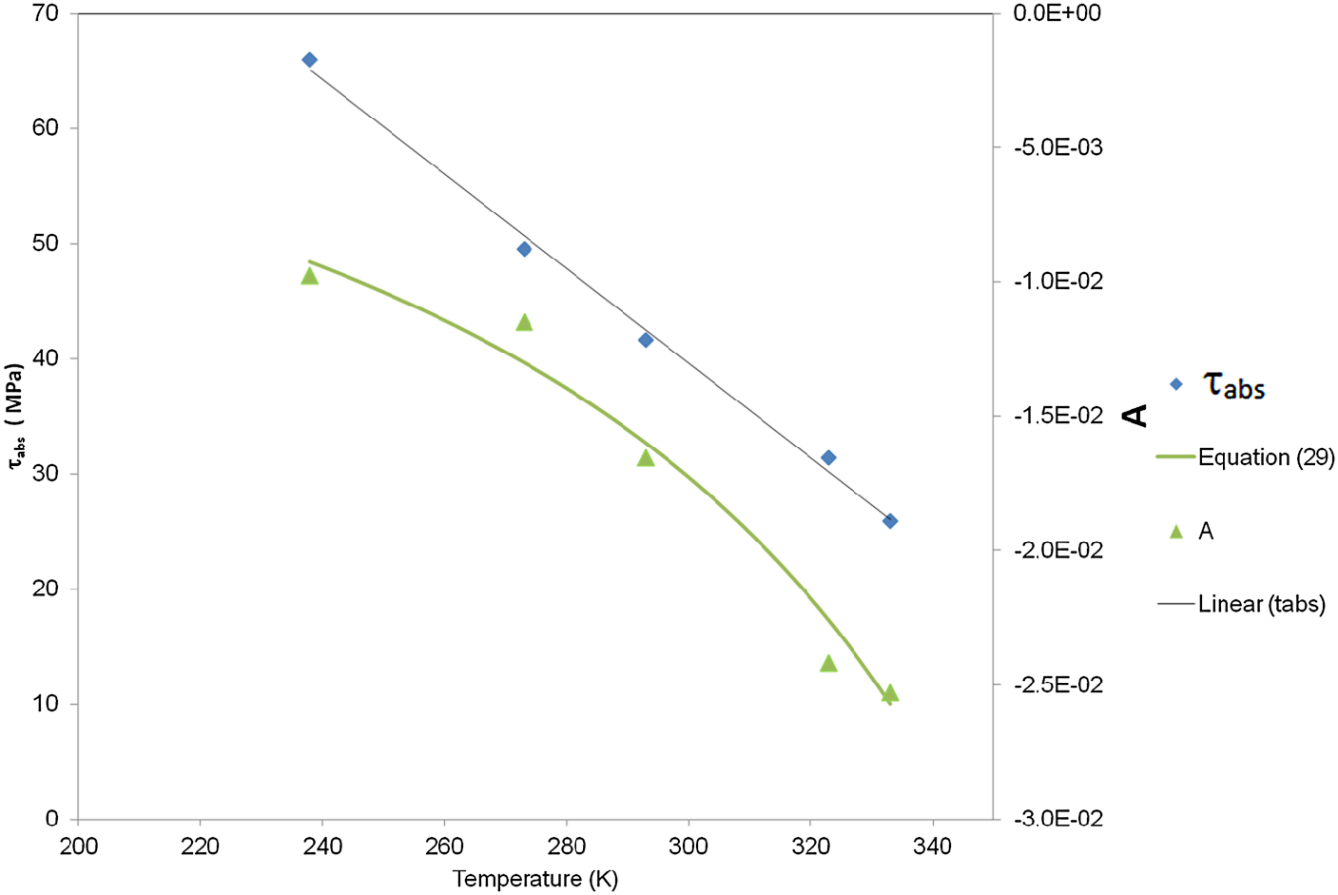
Model parameters as a function of temperature

It was found that satisfactory fits to the experimental data could be obtained while keeping $C$, $D$ and $h$ constant with respect to temperature; these values are shown in Table 1. $h$ was assigned the value of 0.4, the same as that used by Chen and Schweizer (2007a) and considered appropriate for polymethylmethacrylate (PMMA). $D$ was varied between 0 and 0.002 and had only a small ($<1\%$) effect on stress, and was kept at the value 0.0002. The variables $\tau_{\mathrm{abs}}$, $A$ and $C$ were optimized for all temperatures while keeping the remaining parameters constant. The values of $C$ found by this process varied between 1.7 and 2.2 s, and the use of a constant average value, given in Table 1, for all temperatures had an insignificant effect on the yield-stress predictions. With this value of $C$, $\tau_{\mathrm{abs}}$ and $A$ were re-calculated. The values of $A$ were then fitted to Eq. (29) to give values of $P$ and $Q$. Yield-stress predictions are shown in Fig. 3. The values obtained for $\tau_{\mathrm{abs}}$ and $A$ are plotted in Fig. 4, together with the curve from Eq. (29). The constant parameters are given in Table 1. The stresses in Fig. 3 are based on values of $A$ taken from the fitted curve rather than the points; the difference between stress derived using the point values of $A$ rather than the curves is not significant, being at most 2%.

**Table 1:** Model parameters for yield prediction

*C*/s	*D*	*h*	*G*/GPa	*P*	*Q*/K
1.797	2.0 × 10^−4^	0.4	1.0	1.339 × 10^2^	−5.755 × 10^4^

The quality of the fits in Fig. 3 shows that this procedure offers a feasible modeling approach. In effect, the only temperature-dependent parameter is $\tau_{\mathrm{abs}}$, which to a good approximation varies linearly. The model captures the increasing slopes of the curves in Fig. 3 with a single plastic process, comparing favorably with an Eyring representation that would require two or more processes.

## Experimental studies of yield of PET

In order to broaden the scope of the investigation and allow for more detailed study, we have conducted a set of tensile experiments on PET at a series of temperatures. Strain fields and strain rate data have been collected using video extensometry.

### Materials and experimental methods

A commercial grade of PET, Dow Lighter C93, was obtained in the form of granules. According to manufacturer’s specifications this material has a glass transition temperature of 351 K and a melting point of 520 K. The glass transition temperature was further investigated using Modulated Differential Scanning Calorimetry (MDSC) on 5 mg specimens of polymer. A TA Instruments Discovery DSC was used, programmed with a mean temperature ramp of 3 K/min from 273 K (0 °C) to 573 K (300 °C). In order to decouple the reversible heat flow measurements from non-reversible thermal events a modulation amplitude of $\pm1.0~\mbox{K}$ on a period of 1 minute was continuously applied to the samples during heating. The glass transition determined from the reversible signal of the MDSC occurred in the range 343.10–350.95 K, with the upper end of the range in good agreement with the manufacturer’s value.

After drying the granules in a vacuum oven overnight, sheets nominally 0.9 mm thick were compression molded between platens heated to 553 K (280 °C) at a pressure of up to 4 MPa. This method of manufacture is established as one that produces no significant skin-core effects (Kiraly and Ronkay 2013; Jarus et al. 1996). Sheets were then quenched into water at ambient temperature. More DSC measurements were made on the sheet material, using the same instrument as that used above. Specific heats were measured, using a heating rate of 2 K/min and a modulation amplitude of $\pm1.0~\mbox{K}$ on a period of 100 s in the temperature range 273–473 K. For the experimental range 323–346 K, the specific heat was found to vary from $1.15~\mbox{J}\,\mbox{kg}^{-1}$ to $1.49~\mbox{J}\,\mbox{kg}^{-1}$. Crystallinity was calculated from DSC at 2 K/min heating rate by comparing the integrated exotherm of cold crystallization with the melting endotherm, and dividing their difference by the heat of fusion of completely crystalline PET (Kong and Hay 2002). The heat of fusion of crystalline PET was taken as 140 J/g, in accordance with Liangbin et al. (2000), and crystallinity was found to be in the range 9–12%.

Tensile specimens of $33 \times 6~\mbox{mm}$ gauge area (ASTM D638 Type IV 1998) were then cut from the sheets using steel cutters. Grids of dots were printed on the specimen gauge lengths to aid video extensometry, using a MakerBot Replicator 2X 3D printer that had been adapted to hold a permanent felt-tip marker.

Tensile testing was carried out using an Instron 5568 testing machine combined with an environmental chamber. Images of the specimens were taken through the window of the environmental chamber using a PixeLINK model PL-D722MU-T video camera operating at capture rates up to 75 frames/s. After testing, the images were analyzed using the software Fiji, which is based on ImageJ (Schindelin et al. 2012), together with an in-house plugin. The plugin selects triplets of neighboring dots to act as the vertices of constant large-strain triangles, analyzing the triangles exactly to give principal extension ratios. The greatest principal extension ratio is then associated with the axial extension ratio, so that values derived for the latter are independent of the precise orientation of the specimen within the field of view. Testing speeds were in the range 0.54 to 320 mm/min, with testing temperatures 323, 333, 341 and 346 K. Once the environmental chamber had attained the testing temperature, it was held at constant temperature for a 5 minute period before testing.

The adiabatic heating of polymers, which results from the conversion of mechanical energy into heat when they are extended, is a well-established phenomenon (Liu and Harrison 1987) and has been observed specifically in PET (Liao et al. 2015). To assess its significance for our tensile tests, we took thermal images of the specimens using a high-speed infra-red camera. This was done using the tensile testing setup described above. Specimens gripped and were heated to 323 K in the environmental chamber. Since the door of the chamber was opaque to the wavelengths used, it was opened to enable images to be taken while the specimens were extended. A single testing speed of 5.4 mm/min was used. For these measurements an FLIR X6540SC camera operating at 100 frames/s was used.

### Results and analysis: strains

**Fig. 5:**
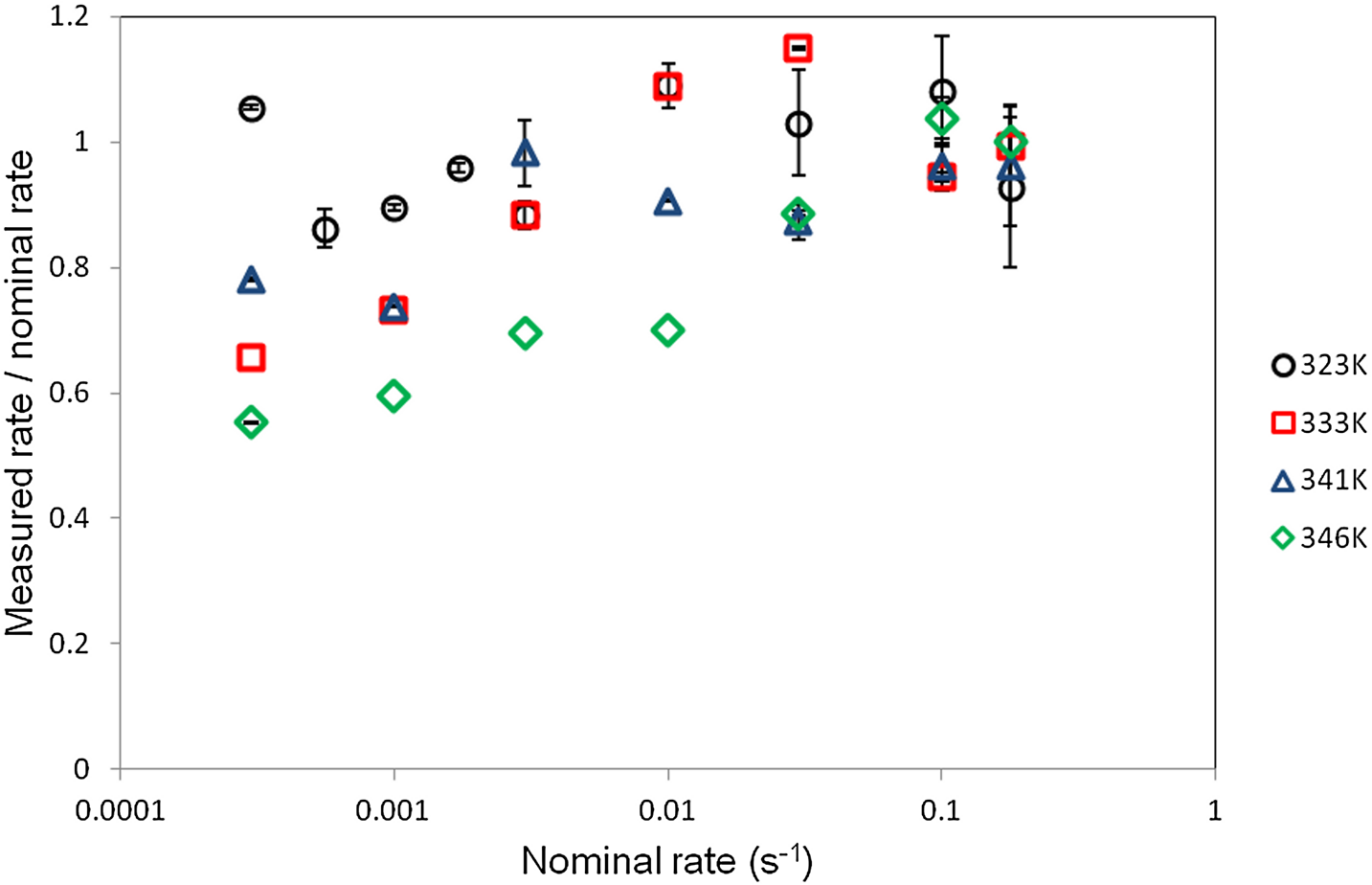
Development of measured strain rate as strain rate increases

In all cases the deformations in the specimen gauge lengths were initially uniform. Depending on the temperature and strain rate, uniform stretching continued until the end of the test, or was followed by shear banding or necking. Strains were measured using image analysis, and this enabled the rates of strain for the uniform stages of deformation to be calculated. These were compared with the nominal rates based on machine speed and on the assumption that all deformation occurs in a specimen gauge length of 30 mm. The measured rates were found to be significantly lower at higher temperatures and low speeds. This is a result of deformation outside the gauge length. At 323 K, the measured rates of strain do not decrease significantly with speed. At higher temperatures, the discrepancy broadly increases with temperature and decreases with testing speed, as shown in Fig. 5, and has essentially disappeared at the highest rate. At the higher rates, the measured strain rates are sometimes somewhat higher than the nominal values, due to the strain being localized into a shorter gauge length than that assumed. When studying the effect of strain rate on yield stress, measured rates are used.

**Fig. 6:**
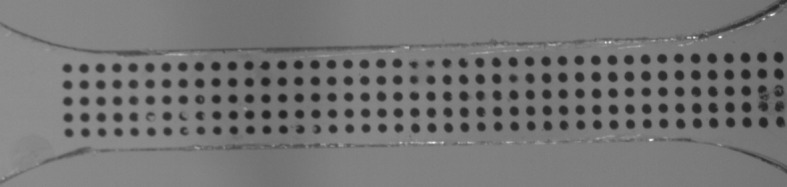
Undeformed gauge length

**Fig. 7:**
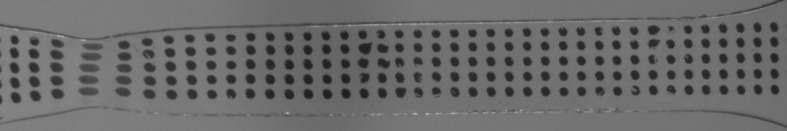
Gauge length deformed at 0.54 mm/min (nominal strain rate $3.0\times 10 ^{-4}~\mbox{s}^{-1}$) to 30% overall nominal strain at 323 K

**Fig. 8:**
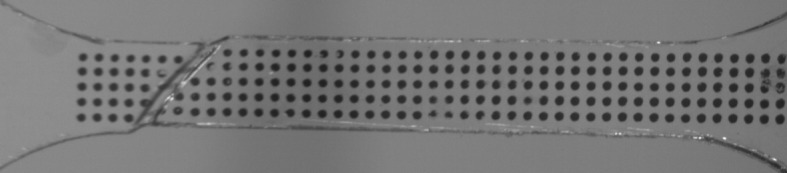
Gauge length deformed at 320 mm/min (nominal strain rate $0.18~\mbox{s}^{-1}$) to 6.5% overall nominal strain at 323 K

At 323 K, instabilities were observed at all testing speeds. In Fig. 6 we show an undeformed gauge length. At low speeds, the shapes of the instabilities are in the form of approximately symmetric necks. In Fig. 7 we show such an instability for this the temperature at a speed of 0.54 mm/min (nominal strain rate $3.0\times 10 ^{-4}~\mbox{s}^{-1}$). At higher speeds the instabilities are in the form of shear bands. Figure 8 shows a fully developed shear band at 323 K for a test at a speed 320 mm/min (nominal strain rate $0.18~\mbox{s}^{-1}$), at an overall nominal strain (specimen extension divided by initial gauge length) of 6.5%; the time elapsed after the start of the test was 0.37 s. The strain at the initiation of banding at this speed varies between experiments over a range 6.5–11.3%. The transition from neck to band occurs between 1.8 and 5.4 mm/min.

**Fig. 9:**
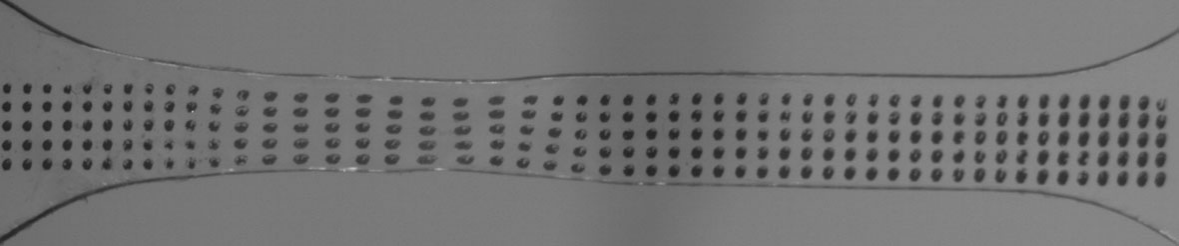
Gauge length deformed at 18 mm/min (nominal strain rate $0.01~\mbox{s}^{-1}$) to 29% overall strain at 333 K

**Fig. 10:**
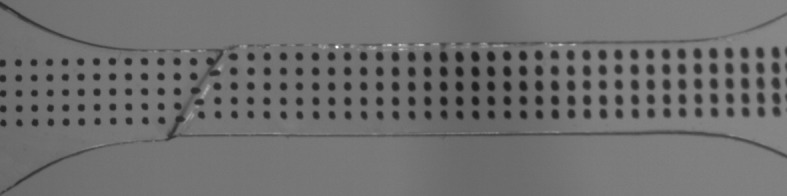
Gauge length deformed at 54 mm/min (nominal strain rate $0.03~\mbox{s}^{-1}$) to 7.4% overall nominal strain at 333 K

At 333 K, the behavior is similar to that at 323 K except that the transition from necking to banding occurs between 18 and 54 mm/min, as shown in Figs. 9 and 10.

At 341 K, strains are uniform at the lowest two speeds 0.54 and 1.8 mm/min (nominal strain rates $3\times 10^{-4}~\mbox{s}^{-1}$ and $10^{-3}~\mbox{s}^{-1}$). At higher speeds up to 54 mm/min necks are observed, whereas bands are observed at 320 mm/min. At 180 mm/min, both necks and bands are observed in nominally identical tests.

**Fig. 11:**
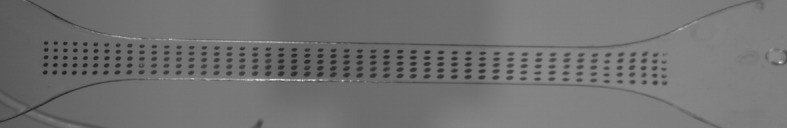
Gauge length deformed at 0.54 mm/min (nominal strain rate $3 \times 10^{-4}~\mbox{s}^{-1}$) to 103% overall nominal strain at 346 K

**Fig. 12:**
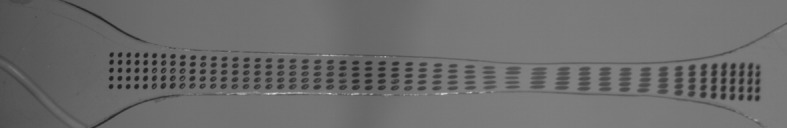
Gauge length deformed at 180 mm/min (nominal strain rate $0.1~\mbox{s}^{-1}$) to 100% overall nominal strain at 346 K

Figure 11 shows the lowest speed test at 346 K, in the glass transition range, where a large uniform deformation is observed. At this the temperature strain remains uniform up to a testing speed of 18 mm/min ($10^{-2}~\mbox{s}^{-1}$) and shallow necks occur at the higher speeds, an example of which is shown in Fig. 12.

### Results and analysis: yield stresses

**Fig. 13:**
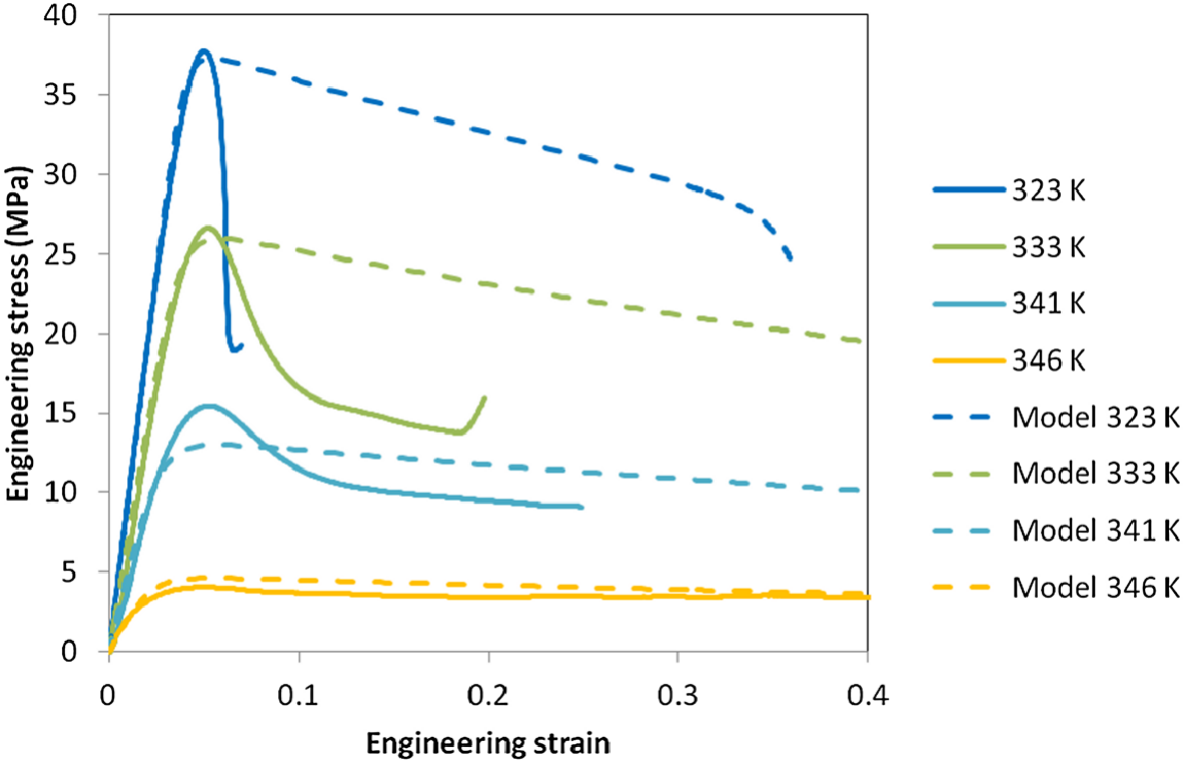
Stress-strain curves at 5.4 mm/min ($3 \times 10^{-3}~\mbox{s}^{-1}$ nominal strain rate) for each temperature, together with predictions from finite element models

Yield stresses can be readily identified. At all temperatures, we observed maxima in engineering stress that we equate with yield stresses. Some examples of stress-strain curves at an intermediate speed are shown in Fig. 13.

**Fig. 14:**
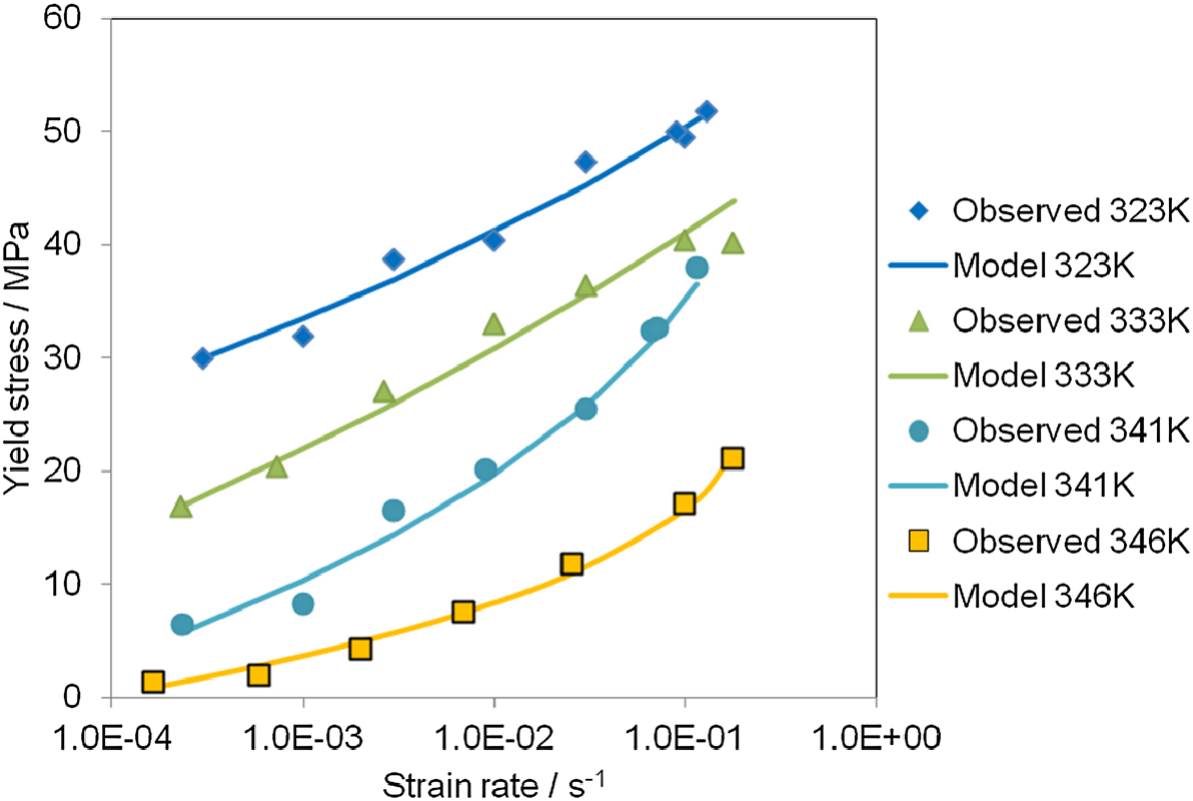
Predictions against experimental data. Model is that defined by Eqs. (9) and (10), with parameter values defined in Table 2

Using the same techniques and software as for the PVC yield data in Sect. 3 above, we have fitted the theory of Sect. 2 to the PET yield data. For these data $h$ was included as a fitted parameter along with $\tau_{\mathrm{abs}}$, $C$ and $A$ while $D$ was kept fixed at $2.0 \times 10^{-4}$ as in Sect. 2. Values of $G$ were obtained from the initial slopes of the stress-strain curves at the highest speeds, using strains measured from the image capture. The parameter values are given in Table 2 and the prediction summarized in Fig. 14.

**Table 2:** Model parameters for yield prediction

Temperature (K)	$\tau_{\mathrm{abs}}$ (MPa)	*C*/s	*A*	*h*	*G* (MPa)
323	42.4	1.435	−0.043	0.53	405
333	43.6	0.692	−0.067	0.47	264
341	42.9	6.313	−0.110	0.25	244
346	16.5	11.14	−0.133	0.30	101

At the temperatures 341 K and 346 K, there is a clear increase in gradient with strain rate, and the model fits reflect this. At the lower temperatures the increasing slope is less obvious, corresponding to the higher values of $h$ which are closer to the value 1 that corresponds to a constant slope and Eyring-like behavior (Chen and Schweizer 2007a). As temperature is increased from 333 to 341 K, the dependence of stress on rate of strain in Fig. 14 becomes stronger, corresponding to a higher strain rate sensitivity and less tendency to instability (Ward and Sweeney 2013). This can be related to the fact that at the lower speeds at this the temperature the deformation is uniform, whereas there are instabilities at all speeds at the lower temperatures. The uniform deformations at 346 K can be attributed to the small drop in stress after the maximum in the stress-strain curve (see Fig. 13), which makes non-uniform deformations less energetically favorable.

**Fig. 15:**
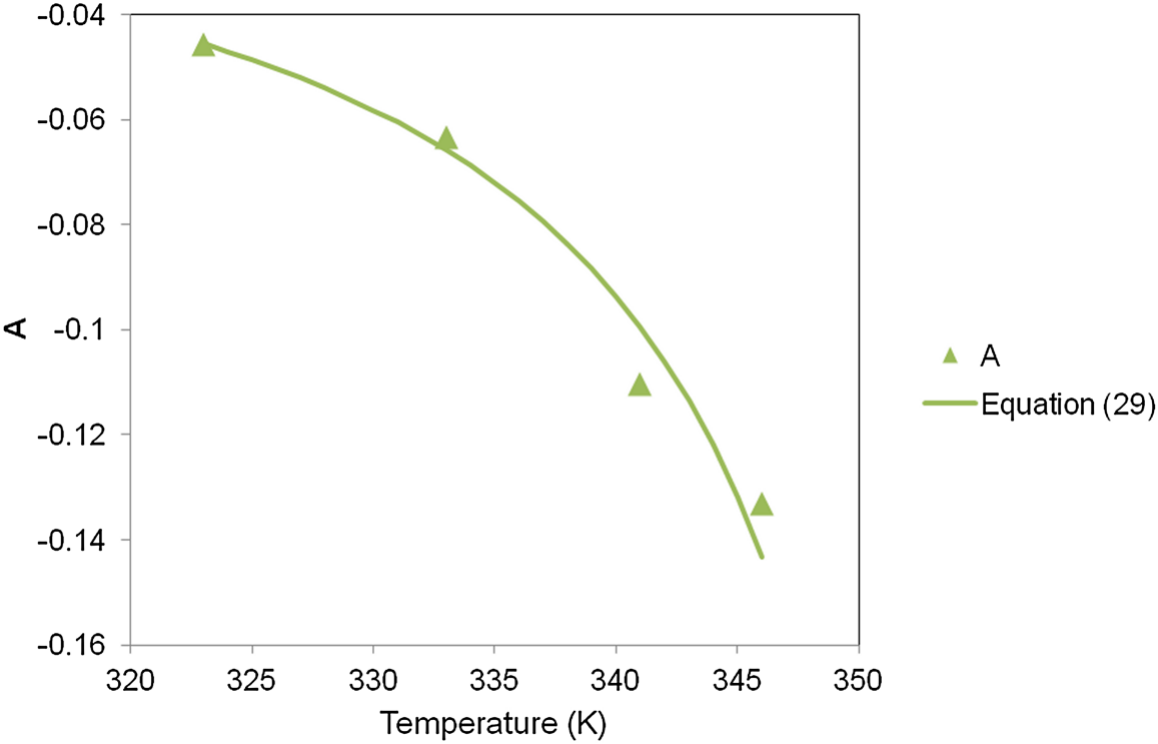
Fitted values of A compared with the analysis using Eq. (29)

We analyze the temperature dependence of the parameter $A$ using the analysis of Sect. 2 and Eq. (29), for which the parameters were found to have values $P = 223.32$ and $Q = - 7.959 \times 10^{4}~\mbox{K}$. The result, shown in Fig. 15, shows a goodness of fit similar to that of Fig. 4 for PVC. Of the other parameters in Table 2, $\tau_{\mathrm{abs}}$ is essentially constant between 323 and 341 K, while C varies unsystematically with temperature. The largest changes for both parameters occur between 333 and 341 K, corresponding to the close approach to the glass transition that starts at 343.1 K.

### Results and analysis: thermal measurements

**Fig. 16:**
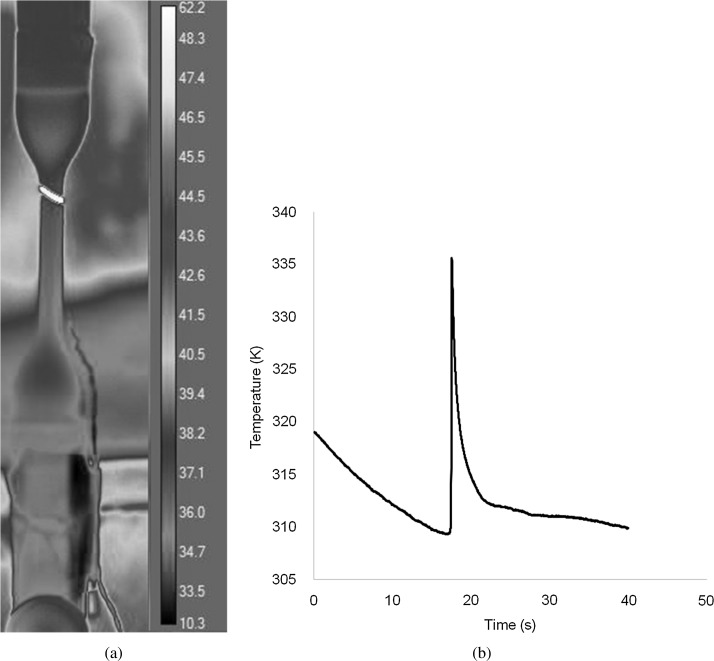
(**a**) Thermal image showing shear band at upper end of gauge length. The scale on the *left* is the temperature in °C. (**b**) Temperature change in band region, beginning at uniform strain and showing a peak coinciding with band formation

In Fig. 16(a) we show a typical thermal image of a stretched specimen while it is in the process of shearbanding. The band is clearly visible as a region of high temperature. Figure 16(b) shows the change with time of temperature averaged over four tests for a single pixel within the region of the instability. The temperature is initially below the set temperature of 323 K as the specimen surface has cooled on opening of the door of the chamber, and it continues to fall until the shear band forms, coinciding with the sharp peak of height 26.3 K.

This the temperature change can be compared with that available from the strain energy. For a peak engineering stress $\sigma_{E}$, the elastic strain energy $W$ is

31 $$ W = \frac{1}{2}\sigma_{E}\alpha \delta , $$

where $\alpha$ is the initial cross-sectional area of the specimen and $\delta$ the extension at the peak engineering stress. The energy H associated with a temperature rise $\Delta T$ is given by

32 $$ H = \rho Vc\Delta T , $$

where $\rho$ is the density, $c$ the specific heat and $V$ the volume. For the 323 K curve in Fig. 13, the stress is seen to fall to approximately half the peak value after shearbanding. This implies that outside the shear band where the material remains elastic, the strain is half its value when in its uniform state just before the band formation. Hence, the deformation in the band can be approximated by $\delta/2$. On this basis we assume that $V = \frac{1}{2}\delta \alpha$ and if we assume that all the strain energy is converted to heat, $H = W$ gives

33 $$ \Delta T = \frac{\sigma_{E}}{\rho c}. $$

For the case 323 K and 5.4 mm/min, Fig. 13 gives a value of $\sigma_{E}$ of 37.7 MPa. For the Dow Lighter material, manufacturer’s data gives a density of $880~\mbox{kg}\,\mbox{m}^{-3}$ and, with c at the measured value for this the temperature of $1.15 \times 10^{3}~\mbox{J}/\mbox{kg}\,\mbox{K}$, we derive a value of $\Delta T$ of 37.3 K. This is in excess of the observed drop, reflecting the expectation that not all the strain energy is converted to heat. This approximate calculation suggests that 70% of the strain energy is converted to heat, which conforms with the range 60–79% found for glassy polymer by Boyce et al. (1992).

## Finite element modeling

The utility of any material model will be limited if it is difficult to implement via the finite element method. In this section we explore this aspect of the model of Fig. 1 as set out in Sect. 2. From the outset it is clear that this theory cannot provide a complete description of polymer behavior. Firstly, we would not expect accurate modeling in post-yield conditions, as all the experimental input data is derived from specimens at and below the yield point. Additionally, the two-dimensional approach ensures that the geometry and stress conditions within a neck or band will not be represented in detail. Also, there is no provision for strain hardening in the model after yield, or stress recovery at constant strain. Both these phenomena can be provided by the addition of a parallel strain-hardening arm in Fig. 1 as first proposed by Haward and Thackray (1968). Furthermore, in the present experiments, the observation of large temperature rises associated with the formation of instabilities can be expected to give rise to serious errors in subsequent predictions. Despite these limitations the model does include some essential features, such as a good description of strain rate dependence and a flow rule that has potential to predict shear banding, and we would expect realistic predictions before the initiation of instabilities. The experimental parameter values of Table 2 are used in the analyses. Two-dimensional plane stress models of the tensile specimens have been created and implemented using the ABAQUS package, with the constitutive model of Sect. 2 implemented using a UMAT user-defined subroutine.

**Fig. 17:**
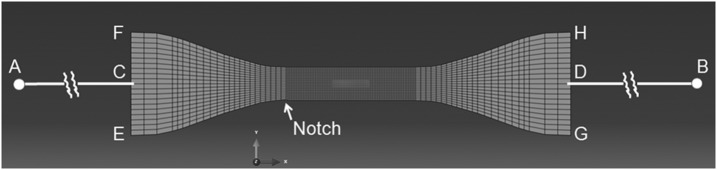
Finite element mesh. The red region defines a set of elements that provide the strain value for the gauge length

The mesh for the ASTM specimen is shown in Fig. 17. Vertical sides FCE and HDG represent the gripped edges. Since the experimental specimens deform asymmetrically on formation of necks or shear bands, a notch is included in the model (marked) by displacing a single node a depth 0.2 mm into the 6 mm gauge length, to enable the deformations to be asymmetric. It is then necessary to include potential for lateral displacement of the specimen; this is done linking the nodes along FCE and HDG by sets of equations that ensure that the two boundaries remain straight, and that their respective normals $AC$ and $DB$ rotate about the points $A$ and $B$. The specimen extensions are applied by fixing $A$ and moving $B$ along the specimen axis while maintaining freedom of rotation of $AC$ about $A$ and $DB$ about $B$, to mimic the experimental loading system. The lengths $AC$ and $DB$ are fixed at 100 mm to correspond to the positions of the pin-joints joining the grips to the Instron load train. The total reaction force is monitored to give access to the engineering stress.

**Fig. 18:**
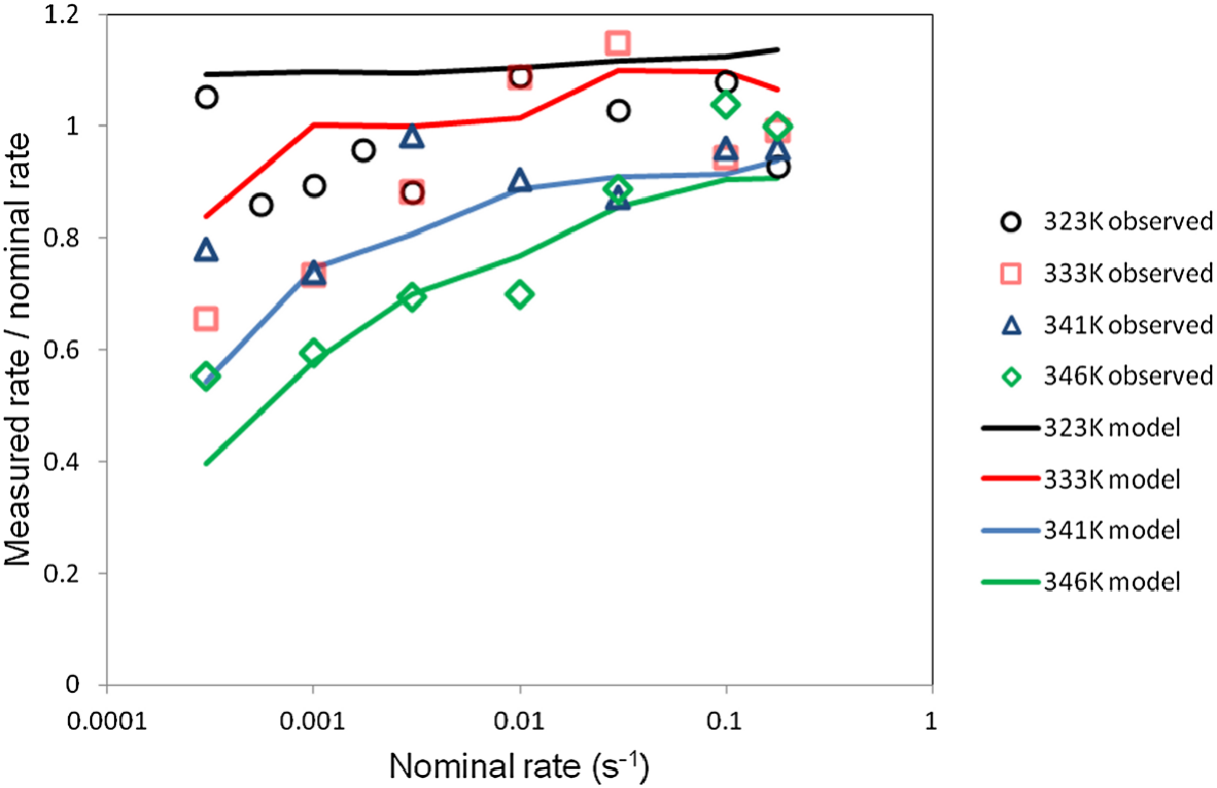
Modeled and observed strain rate in the gauge length

First, the predictions of strain rate at uniform strains will be evaluated. As shown in Fig. 5, strain rate in the gauge length is not simply related to the applied nominal rate. In Fig. 17 we show the finite element model with a set of elements highlighted; over this region the stress and strain are essentially constant and the nodal values are averaged to give values for the strain in the gauge length. In all cases this strain is essentially linear with time after an initial start-up period and before any strain localization occurs, and the strain rate in this linear regime is identified as the strain rate in the gauge length. This is compared with the applied nominal rate by plotting the ratio (rate in gauge length)/(nominal rate) against the nominal rate, as shown in Fig. 18, where it is compared with the experimental ratios. The model and observed ratios show the same trends, with the ratio around unity at 323 K, and increasingly lower than unity as temperature is raised and strain rate lowered. This shows a good level of consistency between the yield-stress behavior, from which we derive the model parameters, and the observed and predicted strains.

**Fig. 19:**
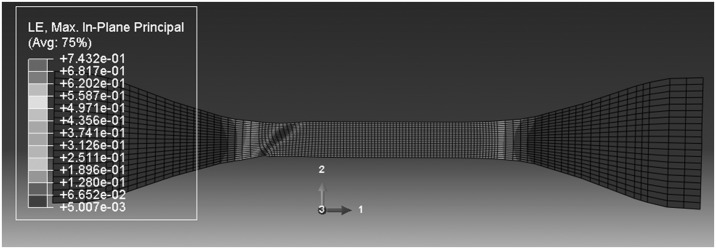
Deformation field at 323 K, 320 mm/min and overall nominal strain 46%

We now turn attention to the deformation field after yield. The possibilities here are that the specimen remains homogeneous or develops an instability in the form of a neck or shear band. As noted previously (Sweeney et al. 2007), a single-arm Maxwell-type model such as that in Fig. 1 has the potential to predict both necks and bands, depending on which of the two processes is responsible for the dominant component of strain. The plastic mechanism, if governed by a Levy–Mises flow rule, will tend to give rise to bands, whereas the elastic component will favor necks. Using the experimentally derived parameter values in Table 2 for 323 K, the finite element models give rise to shear bands at all speeds. This does not conform to experimental observations, as shown in Figs. 7 and 8. The output from a model run at the highest speed of 320 mm/min is shown in Fig. 19. The model strain field bears a qualitative resemblance to the field in Fig. 8, but the model shear band does not begin to form until a nominal strain of 22% is attained, significantly higher than the observed onset of banding at 6.5–11% nominal strain. For comparison, the stress peak corresponding to yield occurs at 6–7% strain experimentally, compared with 6.1% in the model. A delay between the yield point and the band initiation is a feature of both the experiments and the models. The observed temperature increase (Fig. 16) and associated material softening has the potential for both greater instability—a thermal runaway effect—and, by making the elastic mechanism softer, for the formation of a neck rather than a band.

For parameters corresponding to 333 K, the model performance is similar to that at 323 K, with bands predicted rather than necks and the delay between yielding and unstable deformation greater in the model than in the experiments. For both these temperatures Fig. 13 shows that after yield there is a large and rapid drop in observed stress compared with a gradual drop in the model stress, a discrepancy which could be caused by the temperature rises seen in the shear bands.

**Fig. 20:**
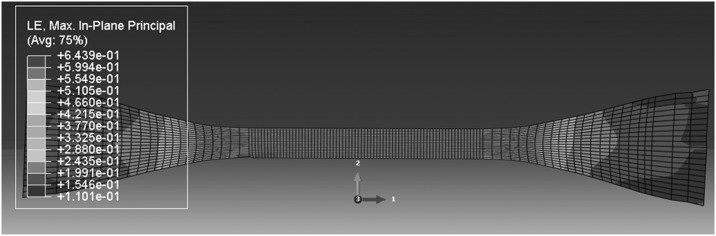
Deformation field at 341 K and 0.54 mm/min, at overall nominal strain 100%

**Fig. 21:**
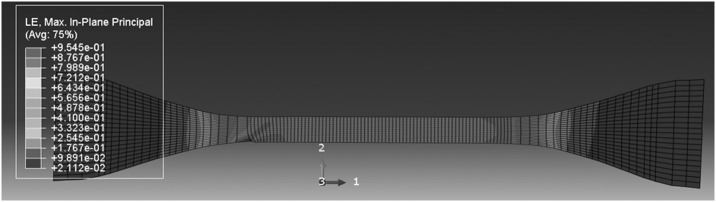
Deformation field at 341 K and 5.4 mm/min, at overall nominal strain 100%, showing beginning of instability

**Fig. 22:**
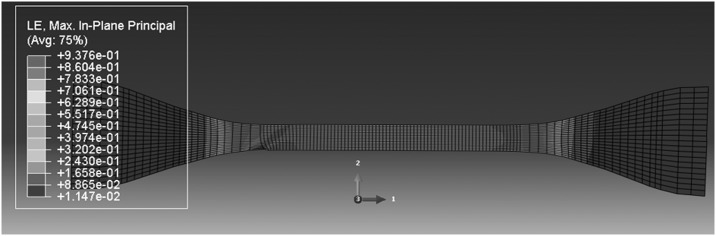
Deformation field at 346 K and 54 mm/min, at overall nominal strain 100%, showing beginning of instability

For the higher temperatures 341 and 346 K simulations, deformations are uniform at the lowest speeds, but bands begin to appear at higher speeds. This is shown in Figs. 20 and 21 for 341 K, where the onset of instability is seen at 5.4 mm/min, as seen in the experiments. At 346 K, the deformation is uniform at 0.54 mm/min, and instability begins at 54 mm/min (see Fig. 22), again in accordance with observation. At these temperatures stresses are lower, so that there is less strain energy, less significant thermal effects and a lower stress drop after the peak. The discrepancies between the shapes of the instabilities—bands in the model and necks in the observation—may be a result of the absence of a strain-hardening mechanism in the model and different strain localization behavior.

The onset of an instability in strain is controlled by the strain rate sensitivity (Ward and Sweeney 2013). In the context of the Chen–Schweizer model, this quantity is the derivative obtained from Eq. (2):

34 $$ \frac{\partial \tau}{\partial \dot{\gamma}} = \frac{\tau_{\mathrm{abs}}}{\dot{\gamma}} \bigl[ - hA \bigl( A\ln ( C\dot{\gamma} ) - D \bigr)^{h - 1} \bigr]. $$

Note that for $h = 1$ the strain rate sensitivity is inversely proportional to the shear strain rate, corresponding to Eyring-like behavior. In general, $\frac{\partial \tau}{\partial \dot{\gamma}} $ decreases with increasing $\dot{\gamma} $. Strain rate sensitivity is a measure of the energy penalty associated with the creation of instabilities, as they are associated with locally high strain rates. Its decrease with increasing strain rate explains why necks or bands may begin to appear as testing speeds are increased, as reflected in both the experiments and the models. However, the model results cannot be expected to give realistic details because of the large experimental temperature changes and absence of strain hardening.

## Discussion and conclusion

Rate-dependent tensile yielding has been modeled to good accuracy by a constitutive model that incorporates a single plastic mechanism as defined by Chen and Schweizer (2007a, 2007b, 2008, 2011) and Riggleman et al. (2008). The yield data include both historical results on PVC and new results on PET. The latter incorporate measurement by video extensometry of the strain fields in the specimen gauge lengths, showing that the strain rate within the gauge length was related to the testing speed in a nonlinear manner.

The model has been implemented as the core of a finite element analysis using a user-defined subroutine and material parameters derived from yield data. Finite element models of the tensile specimens have been created on this basis to give predictions of the strains. The predictions of strain rate compared well with those derived from the strain measurements when the state of strain in the gauge length was uniform. Predictions of non-uniform strain fields were less successful. There are three principal reasons for the discrepancies. First, the model is based on the assumption of isothermal conditions, whereas our observations show the specimens to be highly anisothermal once the strains have become non-uniform. Secondly, the model includes no provision for strain hardening. Finally, the values of the model parameters were derived from yield behavior at uniform strains. The first two are controlling factors for strain localization, which is in many cases observed after yield. To give accurate predictions of instabilities, a constitutive model would need to be thermally coupled; this is independent of whether the core plastic mechanism is that of Chen and Schweizer or some other, such as Eyring’s. The addition of a parallel elastic mechanism could give the required strain hardening. Thus, a more complex model could be developed to give more accuracy. We can conclude that the Chen–Schweizer process can be implemented with no special difficulties and is capable of acting as the core of a constitutive model that returns the principal features of the observed behavior of PET.

The PET yield stresses show dependence on rate of strain that changes with temperature, so that at higher temperatures the slope of the plot of yield stress against the logarithm of rate displays a distinctly increasing slope as rate increases. At lower temperatures the slope is almost constant, corresponding to an Eyring-like behavior. Varying the parameter $h$ with temperature enables the yield predictions to follow this behavior accurately, moving smoothly from Eyring-like characteristics to behavior that would demand two or more Eyring processes.

The present three-dimensional model has been created by causing the Chen–Schweizer mechanism to operate via a flow rule, and adding an elastic mechanism. These appear to be the minimum requirements for a viable model. There is no pressure-dependence of yield. This is a measurable effect in polymers that is customarily incorporated into Eyring-based models using a pressure activation volume term (Ward and Sweeney 2013), and could easily be added to the present model in the same way. More accurately detailed models could be constructed using networks of multiple Chen–Schweizer and elastic mechanisms.
